# Algorithmic recourse in sequential decision-making for long-term fairness

**DOI:** 10.3389/fdata.2026.1750906

**Published:** 2026-02-04

**Authors:** Francisco Gumucio, Lu Zhang

**Affiliations:** Department of Electrical Engineering and Computer Science, University of Arkansas, Fayetteville, AR, United States

**Keywords:** algorithmic recourse, counterfactual, long-term fairness, sequential decision-making, structural causal models

## Abstract

Long-term fairness in sequential decision-making is critical yet challenging, as decisions at each time step influence future opportunities and outcomes, potentially exacerbating existing disparities over time. While existing methods primarily achieve fairness by directly adjusting decision models, in this work, we study a complementary perspective based on sequential algorithmic recourse, in which fairness is pursued through actionable interventions for individuals. We introduce Sequential Causal Algorithmic Recourse for Fairness (SCARF), a causally grounded framework that generates temporally coherent recourse trajectories by integrating structural causal modeling with sequential generative modeling. By explicitly incorporating both short-term and long-term fairness constraints, as well as practical budget limitations, SCARF generates personalized recourse plans that effectively mitigate disparities over multiple decision cycles. Through experiments on synthetic and semi-synthetic datasets, we empirically examine how different recourse strategies influence fairness dynamics over time, illustrating the trade-offs between short-term and long-term fairness under sequential interventions. The results demonstrate that SCARF provides a practical and informative framework for analyzing long-term fairness in dynamic decision-making settings.

## 1 Introduction

Fairness has become a pressing issue in establishing the public's trust in machine learning-driven autonomous systems such as legal, financial, and healthcare (Office of Management and Budget, 2025). Much research has been conducted to alleviate predictive models' discrepancy of treatments toward disadvantaged groups, tackling the issue of fairness via data prepossessing techniques (Biswas and Rajan, 2021; Mroueh et al., 2021), synthetic data generation (Van Breugel et al., 2021; Xu et al., 2019), and the fair batch selection or constraints (Roh et al., 2020). While the majority of the literature focused on the static setting where a predictive model utilizes a fixed dataset to make a single decision, in real-life situations such as bank loans, the model operates in a dynamic setting, making sequential decisions given a history of evolving profile information. Recent studies show that statically addressing fairness does not achieve long-term fairness and may exacerbate discriminatory decisions of predictive models in the long run (Liu et al., 2020; Zhang et al., 2020).

One mainstream line of work in achieving long-term fairness is to learn decision models that incorporate long-term fairness constraints into the learning objective, thereby accounting for the delayed impacts of decisions (for example, by balancing short-term predictive accuracy against long-term equity goals). For instance, Hu et al. (2024) proposes modeling the evolution of features and outcomes over time and embedding long-term fairness constraints into the optimization problem, ensuring that future cohorts are not systematically disadvantaged. Another work by Guldogan et al. (2022) introduces the concept of Equal Improvability, which aims to equalize the potential acceptance rate of rejected individuals across different demographic groups, thereby promoting more equitable outcomes in the long run. Different from these approaches, there has also been growing interest in performative prediction (Perdomo et al., 2020), a framework that highlights how model-dependent distribution shifts arise when individuals or institutions respond to deployed models, and how this framework can be leveraged to achieve long-term fairness (Jin et al., 2024).

In this paper, we investigate long-term fairness from a novel perspective that complements the literature. Rather than achieving long-term fairness through adjusting decision models, we focus on algorithmic recourse, providing actionable recommendations to individuals so that they can improve individuals' qualifications while keeping the decision models unchanged. This problem setting is practically motivated, since individuals naturally seek to improve their outcomes, and many agencies are equipped to offer resources that facilitate such improvement. For example, workforce development programs can provide training courses, career counseling, and mentorship to job seekers who do not meet certain hiring criteria (Holzer, 2015). By participating in these programs, individuals could improve their qualifications, for example, by acquiring relevant skills or certifications (Kasy and Abebe, 2021). By offering structured, feasible recommendations, recourse-based approaches have the potential to mitigate disparities over the long term, as more applicants gain the opportunity to meet or exceed existing acceptance thresholds.

Algorithmic recourse has been extensively studied in static settings, where the goal is to identify minimal feature changes that flip an unfavorable decision to a favorable one (Karimi et al., 2020). Prior work has explored recourse in tabular data (Karimi et al., 2021; von Kügelgen et al., 2022; Dominguez-Olmedo et al., 2022) and image-based domains (Jung et al., 2022; Wang and Vasconcelos, 2020). However, comparatively little is known about recourse in sequential decision-making settings, where individuals interact with a decision system repeatedly over time. Extending recourse to this setting introduces several challenges. First, actions taken at one time step influence future states, feasibility constraints, and available actions. Second, causal dependencies among features, both within and across different time steps, must be explicitly considered to ensure that recommended interventions lead to meaningful and realistic outcomes. Finally, fairness considerations arise not only at individual decision points but also cumulatively over time, raising the risk that myopic recourse strategies may unintentionally amplify long-term disparities.

To address these challenges, we propose Sequential Causal Algorithmic Recourse for Long-term Fairness (SCARF), a causally grounded framework for generating temporally coherent recourse trajectories in sequential decision environments. SCARF is designed as a reference framework rather than an optimal control solution. It integrates: (1) a fixed base classifier; (2) a temporal dynamics module that captures the evolution of individual features; (3) a causal intervention module that models the downstream effects of actions across time steps; and (4) an autoregressive generator that produces feasible, budget-constrained recommendations at each step.

We further provide a theoretical analysis establishing a minimal structural property of sequential recourse: under mild monotonicity and concavity assumptions, distributing intervention effort over time is never worse than deferring all actions to a single final step. This result motivates the need for sequential formulations by demonstrating that single-shot recourse is not a neutral baseline in dynamic settings, which motivates the design choices underlying SCARF.

We conduct experiments on both synthetic and semi-synthetic datasets designed to capture longitudinal changes in individuals' features and outcomes. SCARF is compared against multiple baselines that represent key approaches to algorithmic fairness and recourse. Our results demonstrate that SCARF effectively manages the trade-offs between short-term and long-term fairness, consistently outperforming baseline methods across multiple time steps, while naive extensions of static recourse can lead to compounding unfairness over time. We also perform a sensitivity analysis to assess the robustness of SCARF under varying intervention budgets, along with an ablation study to highlight the critical role of its sequential modeling component.

The contributions of this paper are summarized as follows:

- We formally define the problem of sequential algorithmic recourse under cumulative budget and long-term fairness constraints, extending existing static recourse formulations to dynamic decision-making settings.
- We propose SCARF, a causally grounded reference framework that generates temporally coherent recourse trajectories, serving as a practical reference framework for studying sequential recourse.
- We provide a theoretical analysis establishing a minimal structural property of sequential recourse, showing that temporally distributed interventions weakly dominate deferred single-shot recourse under mild assumptions, which motivates the need for sequential formulations.
- Through empirical evaluation, we demonstrate that naive extensions of static recourse can exacerbate long-term unfairness, while SCARF yields more stable outcomes across time under identical feasibility and budget constraints.

## 2 Related work and preliminaries

### 2.1 Long-term fairness

Most classical work on algorithmic fairness has focused on static, one-shot decisions, but recent studies emphasize that fairness is a dynamic, long-term concern (Liu et al., 2018; D'Amour et al., 2020). To tackle fairness in sequential settings, recent works acknowledge that one must look beyond immediate parity and account for delayed impacts, feedback loops, and temporal dynamics when deploying fair ML systems over time. One line of research embeds fairness constraints into sequential decision-making processes, particularly through reinforcement learning (RL; Jabbari et al., 2017; Wen et al., 2021; Hu et al., 2023; Lear and Zhang, 2025). Another line of research leverages the framework of performative prediction to identify performative feedback loops where deployed predictive models actively shape future data distributions (Perdomo et al., 2020; Jia et al., 2024; Jin et al., 2024). Other research adopts causal frameworks to assess long-term fairness, addressing the evolving impacts of algorithmic decisions from the causal effect perspectives (Hu and Zhang, 2022; Hu et al., 2024).

To complement prior work, we focus on algorithmic recourse, which achieves sequential fairness through actionable interventions rather than altering the decision models themselves. A relevant research is Equal Improvability (Guldogan et al., 2022) which aims to equalize rejected individuals' potential acceptance rates across groups and promote long-term equity. However, Equal Improvability still primarily promotes long-term fairness by modifying the predictive model to equalize opportunities for future improvement. In contrast, SCARF adopts a complementary perspective by studying fairness through personalized recourse recommendations, focusing on how sequential, causally informed interventions unfold over time.

### 2.2 Algorithmic recourse

Algorithmic recourse focuses on providing individuals adversely affected by an automated decision with actionable steps to improve future outcomes. This concept is closely tied to counterfactual explanations that describe how a person could change their features to flip the model's decision (Wachter et al., 2017; Ustun et al., 2019). A notable gap in the literature is how to extend recourse to sequential or temporally evolving scenarios. Most existing recourse frameworks do not account for the possibility that a model is retrained or that an individual's circumstances evolve during the process. Recently, researchers have begun examining the robustness of recourse over time. For instance, Fonseca et al. (2023) study a setting where after a user follows a recourse plan, the decision boundary might shift; they find that ignoring temporal dynamics can lead to invalidated recourse plans that no longer yield a positive outcome by the time they are completed. Another work proposes evaluating and improving the long-term validity of recourse, for example, by incorporating predictive uncertainty or environment trends into the recourse computation (De Toni et al., 2024). However, this area remains largely unexplored. While static recourse techniques are well developed, extending recourse to dynamic, multi-step decision environments remains an open challenge. The proposed SCARF framework provides a principled setting for studying this challenge.

### 2.3 Structural causal models and counterfactuals

Our SCARF framework relies on the formalisms of Structural Causal Models (SCMs) and counterfactual reasoning. An SCM, in the sense of Pearl (2009), consists of a set of random variables linked by directed causal relationships and governed by structural equations. These equations *x*_*i*_: = *f*_*i*_(Pa(*x*_*i*_), *u*_*i*_) specify how each endogenous variable *X*_*i*_ is determined by its parent variables and some exogenous noise *U*_*i*_. By representing cause-effect dependencies explicitly, SCMs allow us to answer interventional and counterfactual queries.

An intervention (via the do-operator) sets a variable to a given value (replacing its structural equation), which lets us compute the effect of that change throughout the system. In this way, one can ask counterfactual questions of the form “What if *X* had been *x*′ instead of *x*?” and obtain the model's implied outcome for some target variable *Y*. This machinery is the backbone of many modern approaches to fairness and recourse. By leveraging structural causal models, SCARF enables the simulation of counterfactual interventions for recourse and the examination of long-term fairness effects under hypothetical changes, providing a causally informed framework for sequential recourse analysis.

## 3 Methods

### 3.1 Problem formulation

#### 3.1.1 Overview

Consider a sequential decision-making process spanning the time range *t* = 1, ⋯ , *T*, involving a group of individuals characterized by a set of profile features **X** and a sensitive feature *S*. At any given time *t*, the profile of an individual is represented by **x**_*t*_. A decision model *h* generates decisions at each step, denoted as *y*_*t*_ = *h*(**x**_*t*_). By default, it predicts ŷ_*t*_ as ŷ_*t*_|**x**_*t*_ = 1 if *h*(**x**_*t*_)≥0.5 and ŷ_*t*_|**x**_*t*_ = 0 otherwise. Subsequently, an unknown transition function τ determines the individual's profile at the following time step based on both the current features and decision, represented as **x**_*t*+1_ = τ(**x**_*t*_, *y*_*t*_). Our framework leverages a temporal dynamics module to learn the evolution of the individual features, as will be detailed in Section 3.2.3.

**Figure 1:**
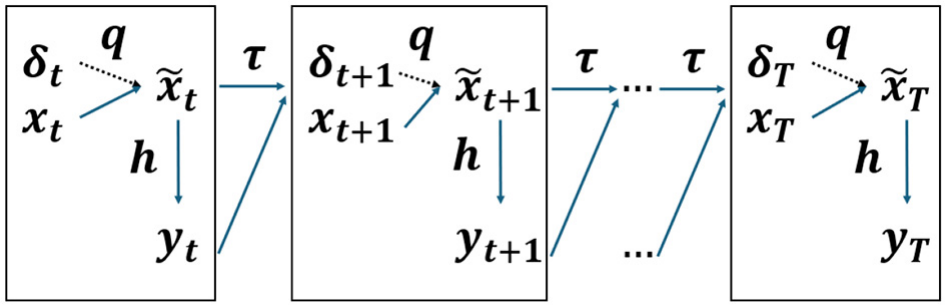
Recourse for sequential decision-making process.

We consider actionable recommendations for each individual that target specific modifiable features at each time step. These recommendations can be represented as interventions δ_*t*_, which, due to the causal relationships among the features, may also influence other related features of the individual. Without loss of generality, we model the effect of such intervention through a function *q* that captures the causal interactions among the features, resulting in counterfactual features ${\tilde{\text{x}}}_{t}=q\left({\text{x}}_{t},\delta_{t}\right)$. For clarity and simplicity, we treat the factual scenario (the scenario without interventions) as a special case where δ_*t*_ = 0. This formulation allows us to uniformly represent both factual and counterfactual scenarios with the same notation, i.e., using **x**_*t*_ contextually to denote either the factual or the counterfactual features (As a special case, we denote the initial distribution before performing interventions as **x**_0_.) Under an intervention scenario, the resulting decision and state transitions become $y_{t}=h\left({\tilde{\text{x}}}_{t}\right)$ and ${\text{x}}_{t+1}=\tau \left({\tilde{\text{x}}}_{t},y_{t}\right)$, respectively. This process is illustrated in Figure 1.

Given the above notation, we formulate the problem of sequential algorithmic recourse for long-term fairness as designing feasible intervention policies that generate personalized recommendations over time, subject to cumulative budget constraints. The formulation explicitly captures both short-term and long-term fairness considerations within a sequential decision-making process, as detailed below.

#### 3.1.2 Intervention

An intervention δ_*t*_ at time step *t* is formulated as a vector specifying modifications to the modifiable features within the individual's profile **x**_*t*_. Specifically, we divide the user profile features **X** into three subsets: (1) **X**_*I*_, the improvable features subject to direct intervention; (2) **X**_*M*_, the features influenced by *x*_*I*_ but not directly intervened; and (3) **X**_*IM*_, the immutable features. Each intervention is thus constrained to **X**_*I*_, with zeros indicating no modification to corresponding features. Practically, interventions should respect feasibility constraints and cost limitations. We define the cost of intervention at time *t* for individual *i* as a function $c\left(\delta_{t}^{i}\right)$, reflecting the real-world cost or effort required to enact the recommendations. As a natural assumption, we assume *c*(0) = 0. To ensure feasibility, the intervention at each step is subject a global budget *B*_*g*_ over the entire population and all time steps: $\forall i,t,\;\overset{T}{\underset{t=1}{\sum }}\underset{i}{\sum }c\left(\delta_{t}^{i}\right)\leq B_{g}$.

**Figure 2:**
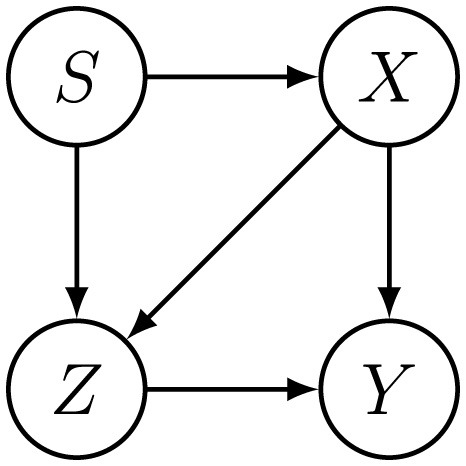
Illustration of causal relationships among features in **X**_*I*_, **X**_*M*_, and **X**_*IM*_ in toy example, where *S* ∈ **X**_*IM*_, *X* ∈ **X**_*I*_, and *Z, Y* ∈ **X**_*M*_.

It is crucial to account for the causal relationships among features when computing counterfactual features under interventions. In general, given an original feature vector **x**_*t*_ and an intervention δ_*t*_, the resulting counterfactual features ${\tilde{\text{x}}}_{t}$ typically differ from the simple sum **x**_*t*_+δ_*t*_, unless all features are causally independent. For example, as illustrated in Figure 2, an intervention on *X* not only alters the value of *X* itself but also affects the values of *Z, Y* due to the causal link between them. As we will describe in detail in Section 3.2.4, our framework integrates a counterfactual inference model to effectively capture these causal effects. When an explicit causal graph is available, this counterfactual inference model can incorporate it directly to enforce graph-consistent counterfactuals. When such knowledge is unavailable, our framework does not assume causal identifiability among features. Instead, it will rely on the intervention constraints **X**_*I*_ and the learned temporal dynamics **x**_*t*+1_ = τ(**x**_*t*_, *y*_*t*_) to produce distributionally plausible counterfactual trajectories.

For improved generalization and effectiveness, we employ an autoregressive process to generate the trajectory of interventions across all time steps based on the initial profile features of individuals. Formally, we represent this process as to learn $P_{\theta }\left(\delta |{\text{x}}_{1}\right)=\Pi_{t=1}^{T}P_{\theta }\left(\delta_{t}|\delta_{<t},{\text{x}}_{1}\right)$, where δ_<*t*_ = (δ_1_, ⋯ , δ_*t*−1_) denotes the sequence of interventions before *t*. This approach allows the model to capture temporal dependencies within the sequential data, thus enhancing the quality and relevance of the generated interventions.

#### 3.1.3 Short-term fairness

Short-term fairness focuses on ensuring equitable decision outcomes at each individual time step, particularly as mandated by regulatory frameworks. To operationalize short-term fairness, we adopt commonly used fairness constraints such as demographic parity or equal opportunity. For example, using demographic parity, we require the decision outcomes to be independent of sensitive feature *S* at each time step:


$$\begin{array}{l} \forall t,\;P\left(h\left({\text{x}}_{t}\right)>0.5|S=1\right)-P\left(h\left({\text{x}}_{t}\right)>0.5|S=0\right)=0. \end{array}$$ (1)

Note that **x**_*t*_ here may represent either factual or counterfactual features depending on the context.

To facilitate continuous optimization, we adopt the approach from Wu et al. (2019) that transforms probabilistic functions into continuous ones. Letting ϕ denote a convex surrogate function, for demographic parity, we obtain:


$$\begin{array}{l} P\left(h\left({\text{x}}_{t}\right)\geq 0.5|s=1\right)-P\left(h\left({\text{x}}_{t}\right)\geq 0.5|s=0\right)\\ =E\left[I\left[h\left({\text{x}}_{t}\right)\geq 0.5|s=1\right]\right]-E\left[I\left[h\left({\text{x}}_{t}\right)\geq 0.5|s=0\right]\right]\\ =E\left[\phi \left(0.5-h\left({\text{x}}_{t}\right)|s=1\right)\right]-E\left[\phi \left(0.5-h\left({\text{x}}_{t}\right)|s=0\right)\right] \end{array}$$

As a result, the loss for short-term fairness $L^{\text{ST}}$ is given by


$$\frac{1}{T}\sum_{t=1}^{T}\left|E[\phi (0.5-h(x_{t})|s=1)]-E[\phi (0.5-h(x_{t})|s=0)\right|$$

#### 3.1.4 Long-term fairness

Long-term fairness refers to equitable outcomes for individuals or groups throughout the extended duration of a sequential decision-making process. Unlike short-term fairness, which assesses fairness at individual decision points, long-term fairness considers how repeated decisions cumulatively impact individuals over multiple time steps, taking delayed effects into account. Consequently, it focuses on aggregated opportunities available to individuals or groups rather than immediate decision outcomes. Consistent with existing literature (e.g., Zhang et al., 2020), we use profile features as metrics to measure individual opportunities and compare the feature distribution across different groups after interventions. Specifically, let $P_{t}^{s}\left(\text{X}\right)$ denote the feature distribution at time step *t* for sensitive group *s*. Long-term fairness is measured by assessing discrepancies between the distributions of different sensitive groups, which captures the cumulative impact of previous decisions on individuals. This is typically done using distributional distance metrics such as Wasserstein distance:


$$\begin{array}{l} W_{p}\left(P_{t}^{s=0}\left(\text{X}\right),P_{t}^{s=1}\left(\text{X}\right)\right)={\left(\underset{\gamma \in \Gamma \left(P_{t}^{s=0}\left(\text{X}\right),P_{t}^{s=1}\left(\text{X}\right)\right)}{\inf}E_{\left(\text{x},{\text{x}}^{\prime }\right)\sim \gamma }\|\text{x}-{\text{x}}^{\prime }{\|}^{p}\right)}^{\frac{1}{p}} \end{array}$$ (2)

where *W*_*p*_ is the *p-Wasserstein distance*, and Γ(ℙ, ℚ) is the set of all *couplings* between distributions ℙ and ℚ. The Wasserstein distance can be computationally expensive for multivariate distributions such as the distributions we are interested in for this work. Due to this, we leverage the commonly used Sinkhorn distance (Cuturi, 2013) as a differentiable and computationally cheaper approximation, denoted by $L^{\text{LT}}$.

#### 3.1.5 Loss function

To construct the training objective, we introduce an improvement loss that encourages the model to shift individuals' predicted outcomes toward favorable predictions (e.g., success, recovery, or improvement), without requiring access to an explicit utility function. This loss is particularly useful in settings where the goal is to increase the proportion of individuals experiencing beneficial outcome changes, independent of sensitive group membership. Concretely, we define the improvement loss as a mean squared error (MSE) between the predicted label *y*_*t*_ after applying the intervention and the target or favorable label *y* = 1.


$$\begin{array}{l} L_{\text{imp}}=\frac{1}{T}\frac{1}{N}\overset{T}{\underset{t=1}{\sum }}\overset{N}{\underset{i=1}{\sum }}{\left(y_{t}^{i}-1\right)}^{2} \end{array}$$ (3)

By combining the above components, we define the following training objective for learning a sequential recourse generator.

**Problem Formulation 1.** *Given a set of trajectories of profile features* X, *where* x∈X *and* **x** = (**x**_1_, …, **x**_*T*_) *represents an individual trajectory, the goal is to learn a generator that produces a sequence of interventions* δ = (δ_1_, …, δ_*T*_) *for each individual by minimizing the objective*


$$\begin{array}{l} \begin{array}{c} L=\lambda_{1}L^{ST}+\lambda_{2}L^{LT}+\lambda_{3}L_{imp}\;s.t.\;\overset{T}{\underset{t=1}{\sum }}\underset{i}{\sum }c\left(\delta_{t}^{i}\right)\leq B_{g}, \end{array} \end{array}$$ (4)

*where* λ_1_, λ_2_, *and* λ_3_ *are balancing coefficients*.

#### 3.1.6 Theoretical analysis

The formulation above characterizes sequential algorithmic recourse under a global budget constraint, where intervention effort may be distributed across multiple time steps. Before introducing the model architecture in the next section, we provide a theoretical analysis to motivate the importance of temporal structure in recourse design. In particular, we examine whether concentrating all intervention resources at a single time point (e.g., the final time step) constitutes a reasonable baseline in dynamic settings. To simplify the analysis, we assume the existence of a gain function *g*(**x**, δ) that quantifies the benefit obtained at each time step given the current features **x** and an intervention δ. In our context, this gain may represent improvements in fairness, reductions in utility loss, or a combination of both. We focus on the cumulative gain accrued over time under different resource allocation strategies. Under mild assumptions, we show that allocating intervention effort over time can yield higher cumulative gain than deferring all resources to a single terminal step. This result establishes a minimal structural property of sequential recourse, demonstrating that single-shot allocation strategies are not a neutral baseline in dynamic environments.

Let *f* denote the composition of the functions τ, *h*, and *q*, defined as


$$f(x_{t},\delta_{t})≜\tau (q(x_{t},\delta_{t}),h(q(x_{t},\delta_{t})))=x_{t+1}.$$

We then have the following proposition.

**Proposition 1**. *Given a sequence of interventions* $\delta ={\left\{\delta_{t}\right\}}_{t=1}^{T}$ *where* $\overset{T}{\underset{t=1}{\sum }}\delta_{t}=B_{g}$ *and* *B*_*g*_ *is the global budget, we have*


$$\overset{T}{\underset{t=1}{\sum }}g\left(f\left({\text{x}}_{t-1},\delta_{t-1}\right),\delta_{t}\right)\geq g\left(f\left({\text{x}}_{1},0\right),B_{g}\right),$$

*i.e., the distributed-over-time resource allocation achieves a larger total gain than the all-at-end strategy, under the following conditions:*

- *g*(**x**, δ) *is concave, monotone non-decreasing in* **x**, δ, *and* *g*(**x**, 0) = 0;
- *f*(**x**, *z*) *is monotone in δ and* *f*(**x**, 0) = **x**.

*Proof*. Let **x**_1_: = *f*(**x**_0_, δ_0_) be the initial state. By mathematical induction, it is easy to show that


$${\text{x}}_{t}=f\left({\text{x}}_{t-1},\delta_{t-1}\right)\geq {\text{x}}_{t-1}\geq {\text{x}}_{1}\;\text{for all }t\geq 2.$$

Because *g* is monotone non-decreasing, we have *g*(*f*(*x*_*t*−1_, δ_*t*−1_), δ_*t*_)≥*g*(*x*_1_, δ_*t*_) for every *t*. Summing over *t* gives


$$\begin{array}{l} \overset{T}{\underset{t=1}{\sum }}g\left(f\left(x_{t-1},\delta_{t-1}\right),\delta_{t}\right)\geq \overset{T}{\underset{t=1}{\sum }}g\left(x_{1},\delta_{t}\right). \end{array}$$ (5)

Then, since *g*(*x*, δ) is concave and *g*(**x**_1_, 0), we have that


$$g\left(x_{1},\lambda B_{g}+\left(1-\lambda \right)0\right)\geq \lambda g\left(x_{1},B_{g}\right)+\left(1-\lambda \right)g\left(x_{1},0\right)=\lambda g\left(x_{1},B_{g}\right).$$

By letting λ = δ_*t*_/*B*_*g*_, it becomes that $g\left(x_{1},\delta_{t}\right)\geq \frac{\delta_{t}}{B_{g}}g\left(x_{1},B_{g}\right).$

Summing this inequality over *t* and noting $\underset{t}{\sum }\delta_{t}=B_{g}$ gives


$$\begin{array}{l} \overset{T}{\underset{t=1}{\sum }}g\left(x_{1},\delta_{t}\right)\geq g\left(x_{1},B_{g}\right) \end{array}$$ (6)

Combining Equations 5, 6 yields


$$\overset{T}{\underset{t=1}{\sum }}g\left(f\left(x_{t-1},\delta_{t-1}\right),\delta_{t}\right)\geq \overset{T}{\underset{t=1}{\sum }}\frac{\delta_{t}}{B_{g}}g\left(x_{1},B_{g}\right)=g\left(x_{1},B_{g}\right).$$

Hence, the proposition is proven.

      □

This result motivates the need for our sequential recourse formulation, as well as the design choices underlying SCARF, as detailed next.

### 3.2 Model architecture

In this section, we present the architecture of Sequential Causal Algorithmic Recourse for Long-term Fairness (SCARF), which operationalizes the sequential recourse formulation introduced in Problem Formulation 1.

#### 3.2.1 Overview

**Figure 3:**
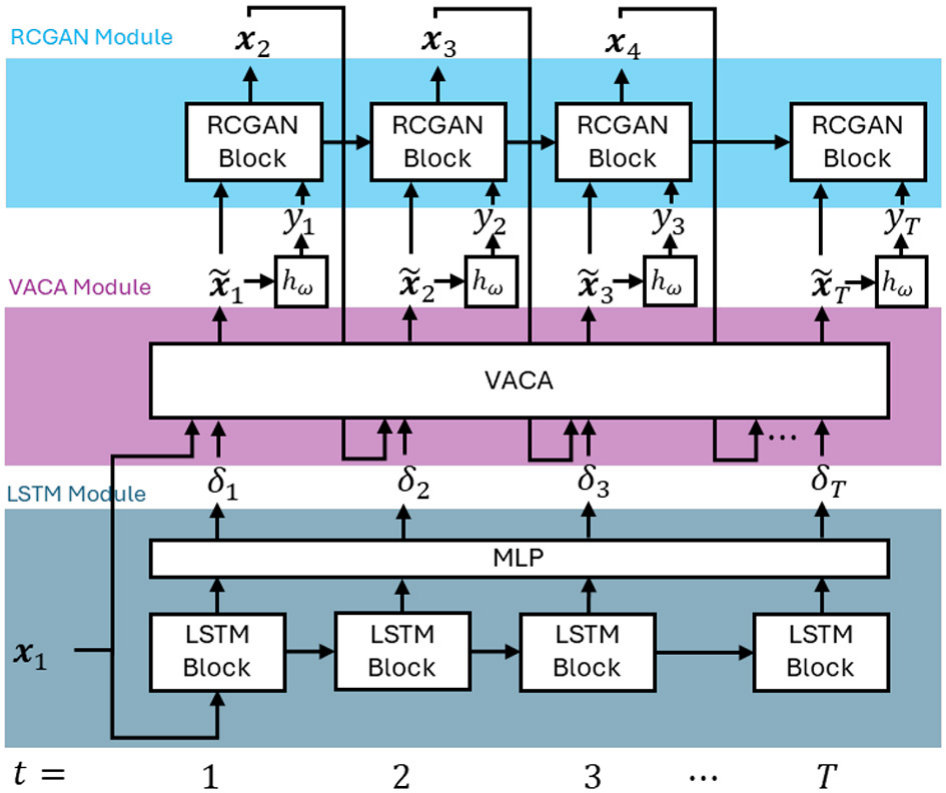
Overview of SCARF architecture.

SCARF is a causality-aware generative framework that maps a trajectory of profile features $\text{x}={\left\{{\text{x}}_{t}\right\}}_{t=1}^{T}$ to a sequence of interventions $\delta ={\left\{\delta_{t}\right\}}_{t=1}^{T}$. The framework is designed to generate feasible, personalized recourse trajectories under cumulative budget constraints, enabling the study of trade-offs between short-term and long-term fairness in sequential decision-making settings, as motivated in Section 3.1. SCARF comprises four main components: (1) a base classifier that produces decisions for each individual at each time step; (2) a temporal dynamics module that captures dependencies across consecutive time steps and models the evolution of individual features; (3) a causal intervention module that applies interventions and generates counterfactual feature trajectories consistent with modeled dependencies; and (4) an autoregressive sequence model that generates intervention sequences conditioned on past states and actions. In our implementation, the temporal dynamics module is instantiated using an adapted Recurrent Conditional Generative Adversarial Network (RCGAN; Hu et al., 2024), the causal intervention module is implemented via an adapted Variational Causal Graph Autoencoder (VACA; Sánchez-Martin et al., 2022), and the intervention generator is realized using a one-to-many LSTM architecture. An overview of the framework is shown in Figure 3. Below, we describe each component in detail and outline the overall training procedure.

#### 3.2.2 Base classifier

The base classifier *h*_ω_(**x**) is built to generate decisions for each individual at every time step based solely on the available profile features **x**. By following conventional fairness guidelines, this classifier explicitly excludes sensitive features from its inputs to prevent direct discrimination. The classifier can be implemented using standard supervised learning methods such as logistic regression, decision trees, or neural networks. Its primary function is to provide consistent decision-making outcomes, which then serve as the baseline scenario upon which interventions and fairness constraints are applied.

During training, the base classifier is independently fitted to observational data. Assuming the decision-making mechanism remains time-invariant, prediction errors are aggregated over all observed time steps. For binary labels $y_{t}^{i}\in \left\{0,1\right\}$ we employ the binary cross-entropy loss, yielding the following training objective:


$$\begin{array}{l} L_{\text{BC}}=-\frac{1}{T}\overset{T}{\underset{t=1}{\sum }}\underset{i}{\sum }E\left[y_{t}^{i}\log{\hat{y}}_{t}^{i}+\left(1-y_{t}^{i}\right)\log\left(1-{\hat{y}}_{t}^{i}\right)\right] \end{array}$$

where ${\hat{y}}_{t}^{i}=h_{\omega }\left({\text{x}}_{t}^{i}\right)$ represents the predicted probability for individual *i* at time step *t*.

#### 3.2.3 Capturing temporal dynamics

To simulate both observational and interventional trajectories of features over time, we adapt the Recurrent Conditional Generative Adversarial Network (RCGAN; Hu et al., 2024) to capture the sequential evolution of feature-label pairs. The RCGAN is trained on full longitudinal observational data, enabling it to model realistic temporal dynamics and support counterfactual generation during inference.

The RCGAN comprises a generator and a discriminator, both implemented using Gated Recurrent Units (GRUs; Cho et al., 2014). This recurrent structure allows the model to capture both temporal and causal dependencies across time steps. The generator is conditioned on the sensitive feature *s*, a sequence of noise vectors ${\left\{u_{t}\right\}}_{t=1}^{T-1}$, the observed feature sequence ${\left\{{\text{x}}_{t}\right\}}_{t=1}^{T}$, and the base classifier *h*_ω_. The hidden state is initialized via a multilayer perception (MLP) applied to the feature vector at the first time step, i.e., **h**_1_ = MLP(**x**_1_). Then, at each of the following time step *t*, the generator input is constructed by concatenating the previous predicted label ŷ_*t*−1_, the sensitive feature *s*, and the noise vector *u*_*t*−1_, yielding **v**_*t*−1_ = [ŷ_*t*−1_, *s, u*_*t*−1_]. This input is fed into the GRU to update the hidden state: **h**_*t*_ = GRU(**v**_*t*−1_, **h**_*t*−1_), which is then used to produce the next feature vector via another MLP: ${\hat{\text{x}}}_{t+1}={\text{MLP}}^{\prime }\left({\text{h}}_{t}\right)$. The predicted label at the next step is computed via the base model as ${\hat{y}}_{t+1}=h\left({\hat{\text{x}}}_{t+1}\right)$. The discriminator receives entire trajectories of generated features and is trained to discriminate between real (observed) and synthetic (generated) sequences. It outputs a value of 1 for real sequences and 0 for generated ones at the sequence level.

To train the RCGAN, we optimize a composite objective that combines the classical adversarial loss with a Maximum Mean Discrepancy (MMD) loss (Gretton et al., 2006) to encourage alignment between the distributions of real and generated sequences. Specifically, the discriminator is trained to maximize:


$$\begin{array}{l} \underset{\phi }{\max}L_{d}\left(\phi \right)=\;E_{\text{X}}\left[\log D_{\phi }\left(\text{X}\right)\right]+E_{\text{Z}}\left[\log\left(1-D_{\phi }\left(G_{\psi ,\omega }\left(S,\text{U},{\text{X}}_{1:T}\right)\right)\right)\right] \end{array}$$ (7)

while the generator is trained to minimize:


$$\begin{array}{r} \underset{\psi }{\min}L_{g}\left(\psi \right)=E_{\text{Z}}\left[\log\left(1-D_{\phi }\left(G_{\psi ,\omega }\left(S,\text{U},{\text{X}}_{1:T}\right)\right)\right)\right]\\ +\gamma \cdot \text{MMD}\left(\text{X},G_{\psi ,\omega }\left(S,\text{U},{\text{X}}_{1:T}\right)\right) \end{array}$$ (8)

Here, *D*_ϕ_ denotes the discriminator, *G*_ψ, ω_ is the generator conditioned on the base classifier *h*_ω_, and γ is a regularization coefficient that controls the strength of the MMD alignment. This formulation enables RCGAN to not only reproduce realistic observational trajectories but also generalize to plausible counterfactual scenarios under a wide range of interventions.

#### 3.2.4 Generating counterfactual features

To compute the causal effects of interventions at each time step, we adapt the Variational Causal Graph Autoencoder (VACA; Sánchez-Martin et al., 2022), a generative model designed to capture how interventions propagate along structured causal pathways. In our framework, VACA is trained on the semi-synthetic temporal data produced by the RCGAN, which models realistic feature trajectories under observational dynamics. This combination allows VACA to learn causal effects within the temporal context established by RCGAN-generated sequences.

**Figure 4:**
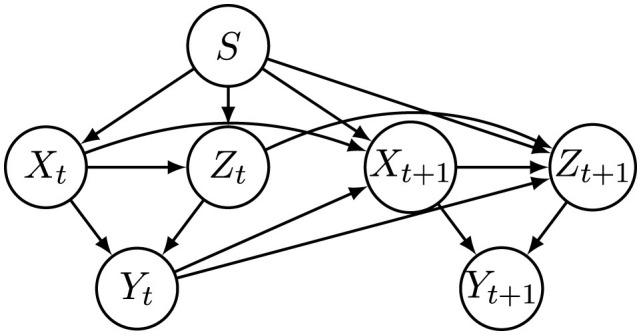
Window causal graph corresponding to causal graph in Figure 2.

To explicitly capture temporal causal relationships, the VACA model operates on a windowed causal graph *G*, which encodes directed dependencies among variables across multiple time steps within a specified time window. This causal graph can either be provided in advance based on domain knowledge or estimated through structure learning methods. Leveraging this causal structure, VACA guides the generation of counterfactual features by controlling information flow within the latent representation space, thus ensuring causal consistency across time steps. Figure 4 illustrates an example of a windowed causal graph derived from the local causal relationships depicted in Figure 2, where only the first-order temporal causal dependency is considered. Specifically, the local causal structure shown in Figure 2 is replicated at each time step, while additional temporal links are introduced to reflect that each variable's value depends both on its own previous value and on the decision made at the preceding time step. In practice, our method can be adapted to accommodate more complex temporal causal dependencies.

The VACA consists of an encoder-decoder architecture that maps observed data into a latent space, respecting the causal structure defined by the windowed causal graph. The encoder infers latent representations by conditioning each variable's latent encoding on its causally related predecessors according to the causal graph. Correspondingly, the decoder reconstructs observed data from these structured latent representations. During training, VACA optimizes a variational evidence lower bound (ELBO), comprising a reconstruction loss and a Kullback-Leibler divergence regularization term as shown below:


$$\begin{array}{l} L_{\text{VACA}}=\\ E_{q_{\phi }\left(\text{z}|{\text{x}}_{t-1},{\text{x}}_{t},\delta_{t}\right)}\left[\log p_{\theta }\left({\text{x}}_{t}|\text{z},\delta_{t}\right)\right]-\text{KL}\left(q_{\phi }\left(\text{z}|{\text{x}}_{t-1},{\text{x}}_{t},\delta_{t}\right)\|p\left(\text{z}\right)\right) \end{array}$$ (9)

where *q*_ϕ_(**z**|**x**_*t*−1_, **x**_*t*_, δ_*t*_) denotes the encoder that infers a latent representation from the observed transition and intervention, and *p*_θ_(**x**_*t*_|**z**, δ_*t*_) is the decoder that reconstructs the intervened state using the latent code and the causal graph.

At test time, for each time step *t*, VACA receives the current features **x**_*t*_, the preceding features **x**_*t*−1_, the intervention vector δ_*t*_ specifying changes to a subset of variables, as well as the window causal graph *G*. It enables counterfactual generation by applying the intervention δ_*t*_ to the observed features **x**_*t*_, conditioned on **x**_*t*−1_. The resulting counterfactual ${\tilde{\text{x}}}_{t}=\text{VACA}\left({\text{x}}_{t},\delta_{t}|{\text{x}}_{t-1}\right)$ can then be passed to a downstream classifier and recursively fed into the RCGAN to simulate forward trajectories under intervention. This enables fairness evaluation and recourse planning based on the underlying causal mechanisms.

#### 3.2.5 Recourse

Finally, to generate individualized and temporally coherent intervention policies, we employ a sequence model that produces intervention strategies for each individual based on their initial feature vector in an autoregressive manner. The objective is to balance improvements in predictive outcomes with fairness considerations across both short- and long-term horizons. Once trained, the model can be applied independently to generate interventions for new instances. For simplicity, we adopt a Long Short-Term Memory (LSTM) network in this work, which autoregressively generates a sequence of interventions over *T* time steps. In practice, other sequence modeling architectures like transformers can also be considered.

The LSTM model, parameterized by η, takes as input the initial feature vector **x**_0_ of an individual and generates a sequence of intervention vectors {δ_1_, δ_2_, …, δ_*T*_}, formally expressed as {δ_1_, δ_2_, …, δ_*T*_} = LSTM_η_(**x**_0_). Although the input comprises only the initial features, the recurrent architecture of the LSTM ensures that each subsequent intervention at time *t* benefits from the evolving internal hidden states, which encode dependencies across previous interventions.

During training, we integrate the previously trained RCGAN and VACA modules into the overall framework to compute the loss according to Equation 4. Specifically, given a new individual, the LSTM first generates a sequence of interventions which are then passed through the VACA model to simulate their immediate causal effects, and subsequently through the RCGAN to model the resulting future feature trajectories. The final loss is computed based on these generated trajectories. During this procedure, the parameters of the RCGAN and VACA modules are kept fixed, and only the LSTM parameters are updated. Constraints for budget limitations are enforced through hard-projection techniques applied during optimization.

## 4 Experiments

In this section, we present experimental results to empirically study the behavior of the proposed SCARF framework in sequential recourse settings. We compare SCARF with relevant baselines on synthetic and semi-synthetic datasets, with the goal of examining how different recourse strategies affect long-term outcomes and fairness when interventions are applied over time.

### 4.1 Datasets and metrics

Our experiments utilize both synthetic and semi-synthetic datasets designed to capture longitudinal changes in individuals' features and outcomes. Longitudinal data is crucial for evaluating the cumulative impact of personalized interventions across multiple decision steps. However, standard fairness-related datasets typically lack this structure. For instance, the widely used datasets derived from the American Community Survey (ACS) Public Use Microdata Sample (PUMS) in Ding et al. (2021) are cross-sectional, i.e., individuals sampled annually are not tracked over multiple periods, preventing meaningful evaluation of interventions. Therefore, following recent practices from Hu et al. (2024), we construct synthetic and semi-synthetic datasets suitable for sequential fairness evaluations, as detailed below.

#### 4.1.1 Synthetic dataset

. We generate a synthetic dataset guided by the causal structure depicted in Figure 2. For the initial distribution, we construct separate multivariate normal distributions over *X* and *Z* for the disadvantaged and advantaged groups, identified by the sensitive attribute *S*. Samples drawn from these distributions form the initial dataset, with the true label *Y* assigned through a Bernoulli distribution.

To create feature values at subsequent time steps (*t*>1), we adopt the following generation rule, reflecting causal and temporal dependencies illustrated in Figure 4:


$$X^{t+1}=\{\begin{array}{l} X_{t}-\epsilon \cdot \beta +b,\hat{Y}=1,Y=0\\ X_{t}+\epsilon \cdot \beta +b,\hat{Y}=1,Y=1\\ X_{t}+b,\hat{Y}=0 \end{array}$$ (10)

where parameter ϵ controls the magnitude of updates; *b* = *S*·*b*_1_+(1−*S*)·*b*_0_ encodes group-specific drift; and β is derived from the ground-truth model *h*_β_. These parameters are systematically varied across experiments to assess robustness under scenarios of varying levels of inherent unfairness, as demonstrated in the sensitivity analysis in the next subsection.

#### 4.1.2 Taiwan dataset

**Figure 5:**
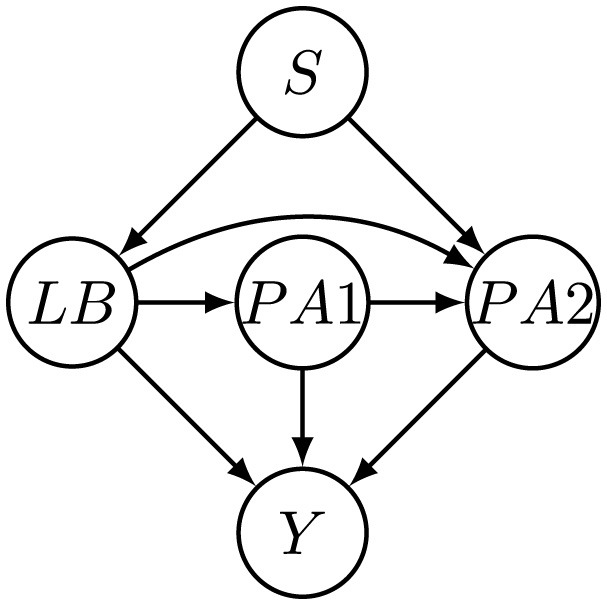
Causal graph of the initial distribution of the Taiwan dataset.

**Figure 6:**
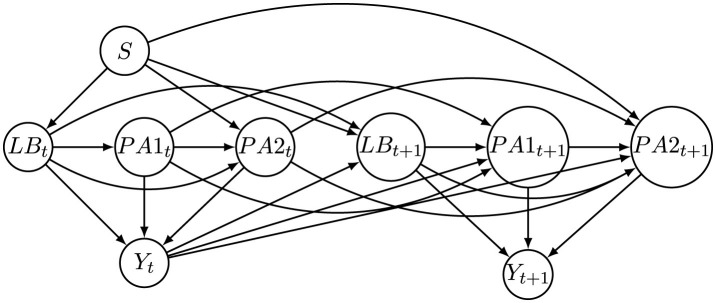
Window causal graph of the Taiwan dataset.

We also construct a semi-synthetic dataset by using real-world data from the Taiwan credit dataset introduced by Yeh and Lien (2009), which contains credit-related features used to predict credit card default. Following the methodology in Hu et al. (2024), we extract three continuous features: Limit Balance (LB), the last payment amount (PA1), and the previous payment amount (PA2), as well as the sensitive attribute *S* and the decision label *Y*, to form the initial distribution at *t* = 1. We employ the BFCI algorithm (Colombo et al., 2014) to learn a causal graph among these features, as depicted in Figure 5. Subsequent time steps (*t*>1) are generated by applying rules analogous to Equation 10. Specifically, each feature at time *t*+1 depends on its own value at time *t*, its causal parents at time step *t*+1, the sensitive attribute *S*, and the decision outcome at time step *t*, thereby capturing realistic temporal and causal dynamics as shown in Figure 6.

#### 4.1.3 Evaluation metrics

We evaluate performance using three primary metrics: short-term fairness is quantified using demographic parity, which measures the independence between sensitive attributes and model decisions at each decision-making step, as shown in Equation 1; long-term fairness is evaluated using the Wasserstein distance, which assesses discrepancies between the distributions of different sensitive groups over multiple time steps, as shown in Equation 2; and utility is measured through prediction accuracy, reflecting the effectiveness of the decision model in maintaining predictive performance.

### 4.2 Implementation of SCARF

We implement SCARF by integrating four components into a unified pipeline: a fully connected Base classifier (two ReLU-activated hidden layers), an LSTM-based intervention generator (two layers, 128 hidden units, dropout 0.2), the VACA causal simulation module (encoder-decoder with latent size 32), and the RCGAN temporal modeling module (generator and discriminator with two layers of 128 units each). Window causal graphs shown in Figures 4, 6 are used for the synthetic and Taiwan datasets, respectively.

SCARF is trained in a sequence of the base classifier, RCGAN, VACA, then the LSTM-based generator. Hyperparameters are selected via grid search on a validation set. The configuration yielding the best validation performance is used for all subsequent evaluations. In both datasets SCARF is trained using 700 samples and validated with 125 samples. Approximated runtime for one setting is 1 h in both datasets. For each setting, we report the average performance for 200 samples over 30 independent runs. All experiments are conducted on NVIDIA Tesla V100 GPUs.

### 4.3 Baselines

We compare SCARF against several prominent baselines from the fairness literature.

Demographic parity (DP; Dwork et al., 2012): a fairness-constrained logistic regression classifier that enforces equal positive prediction rates across sensitive groups, without generating interventions.

Equal improvability (EI; Guldogan et al., 2023): a method that provides interventions to equalize the probability of individuals previously rejected crossing the decision boundary.

Bounded effort (BE; Heidari et al., 2019): generates interventions at each step to assist rejected individuals, with a predefined cap on the total effort.

Effort-based recourse (ER; Gupta et al., 2019): minimizes disparities between sensitive groups in terms of the average effort required to achieve positive outcomes.

Individual-level fair causal recourse (ILFCR; von Kügelgen et al., 2022): aims to ensure individual-level fairness by equalizing the minimal average effort required across sensitive groups for achieving positive outcomes.

To maintain consistency across evaluations, the baseline methods also leverage the RCGAN for generating the sequential data. When recourse actions are involved, VACA is used to compute counterfactual features across all applicable methods. For DP and EO methods, no explicit interventions are simulated by VACA, as these approaches solely focus on fairness constraints in predictions. All baselines are trained independently and evaluated under identical experimental conditions, intervention budgets, and temporal constraints as SCARF. Specifically, the total intervention budget is fixed and distributed across time steps to match the budget conditions applied to SCARF.

## 5 Results and discussion

In this subsection, we present experimental evaluations to assess the performance of SCARF against baseline methods. Our analysis focuses on three questions: (1) how SCARF balances long-term and short-term fairness; (2) how sensitive SCARF's performance is to variations in budget constraints; and (3) the role of the LSTM-based intervention generator through an ablation study.

### 5.1 Evaluating trade-off between long-term and short-term fairness

**Figure 7:**
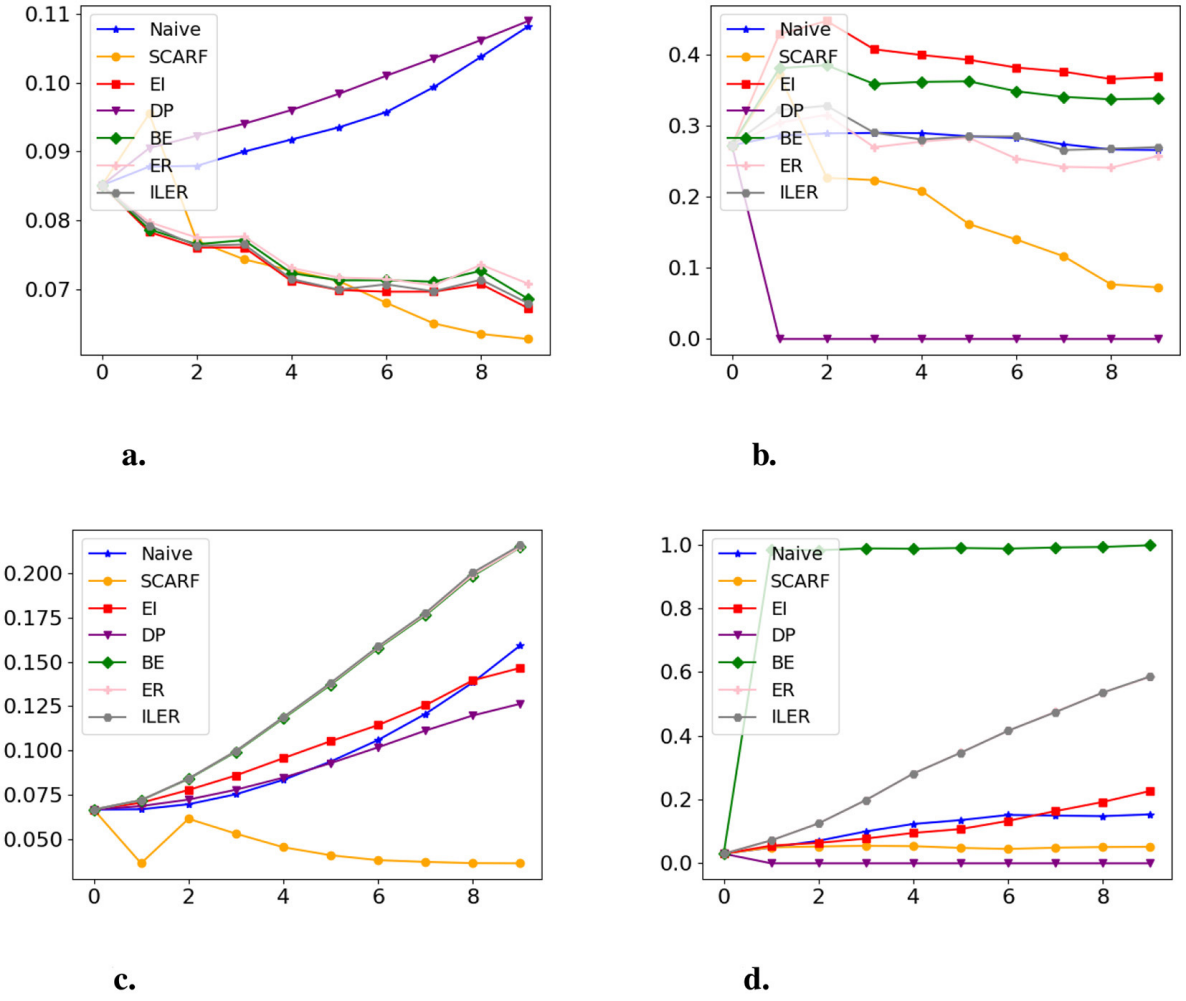
Long-term and short-term fairness results for all methods on the synthetic dataset (budget = 20) and the Taiwan dataset (budget = 30). X-axis: time step; Y-axis: fairness discrepancy (lower is better). **(a)** Synthetic dataset, long-term. **(b)** Synthetic dataset, short-term. **(c)** Taiwan dataset, long-term. **(d)** Taiwan dataset, short-term.

We first evaluate the empirical trade-off between long-term fairness and short-term fairness. Figure 7 compares SCARF to baseline methods. For the synthetic dataset (Figures 7a, b), SCARF exhibits a pattern in which long-term fairness disparities are reduced over time while maintaining bounded short-term fairness deviations. While SCARF demonstrates slightly higher short-term fairness discrepancies initially compared to the DP method that is explicitly designed to enforce immediate fairness, its performance improves progressively over time, ultimately approaching the short-term fairness of DP by the end of the evaluation period. In contrast, DP achieves strong immediate fairness but shows rapidly increasing long-term disparities. On the other hand, baselines explicitly optimized for long-term fairness (e.g., EI and ILFCR) often show weaker short-term fairness across the time steps. These results illustrate how different recourse strategies induce distinct temporal fairness dynamics, with SCARF finding a relative balance between short-term and long-term fairness.

Results on the Taiwan dataset show qualitatively similar trends. As shown in Figure 7c, SCARF is associated with lower long-term fairness disparities relative to most baselines, while maintaining comparatively stable short-term fairness across time steps (Figure 7d). These observations suggest that the sequential structure of SCARF enables more balanced temporal fairness dynamics in a realistic setting, without enforcing fairness exclusively at a single time scale.

We also report the prediction accuracy of SCARF and compare it to that of EI for the synthetic dataset, where we have the ground-truth decision model to evaluate accuracy at all time steps. Note that, unlike EI, SCARF does not alter the underlying classifier, thereby better preserving predictive utility. As expected, the average prediction accuracy of SCARF over 10 time steps is 0.86 ± 0.01, which exceeds that of EI, reported as 0.84 ± 0.04.

### 5.2 Sensitivity analysis

**Figure 8:**
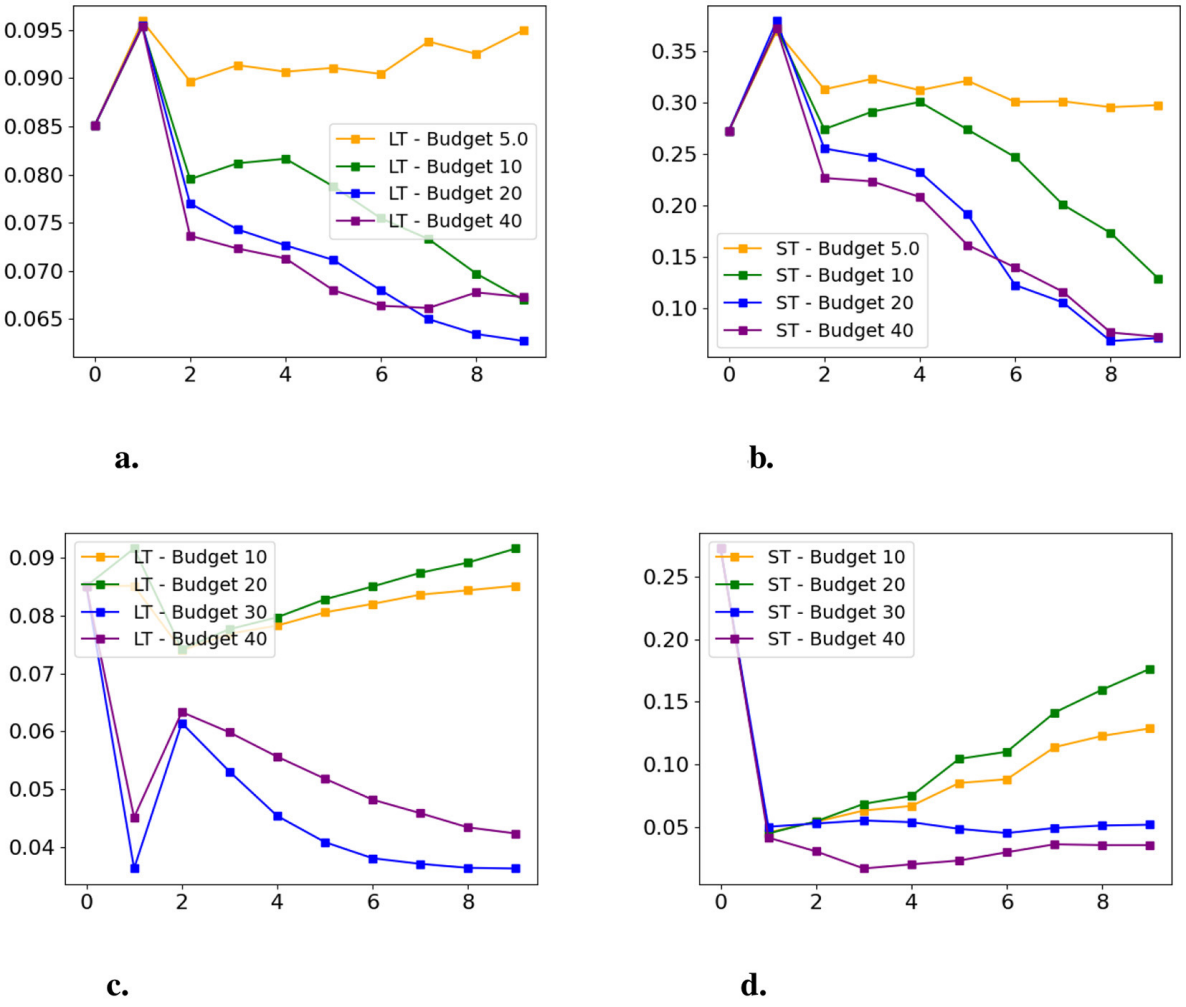
Budget sensitivity analysis on both datasets. X-axis: time step; Y-axis: fairness discrepancy (lower is better). **(a)** Synthetic dataset, long-term. **(b)** Synthetic dataset, short-term. **(c)** Taiwan dataset, long-term. **(d)** Taiwan dataset, short-term.

We next examine SCARF's sensitivity to variations in intervention budgets on both datasets. Figure 8 summarizes the fairness discrepancy results under different budgets. As can be seen, increasing the budget consistently improves long-term fairness, as shown by progressively lower fairness discrepancies at later time steps. A similar positive effect of budget increase is also observed in short-term fairness, where higher budgets correspond to a gradual reduction in short-term disparities as the sequence progresses. These results illustrate how budget constraints shape the temporal dynamics of fairness in sequential recourse settings.

### 5.3 Ablation study

**Figure 9:**
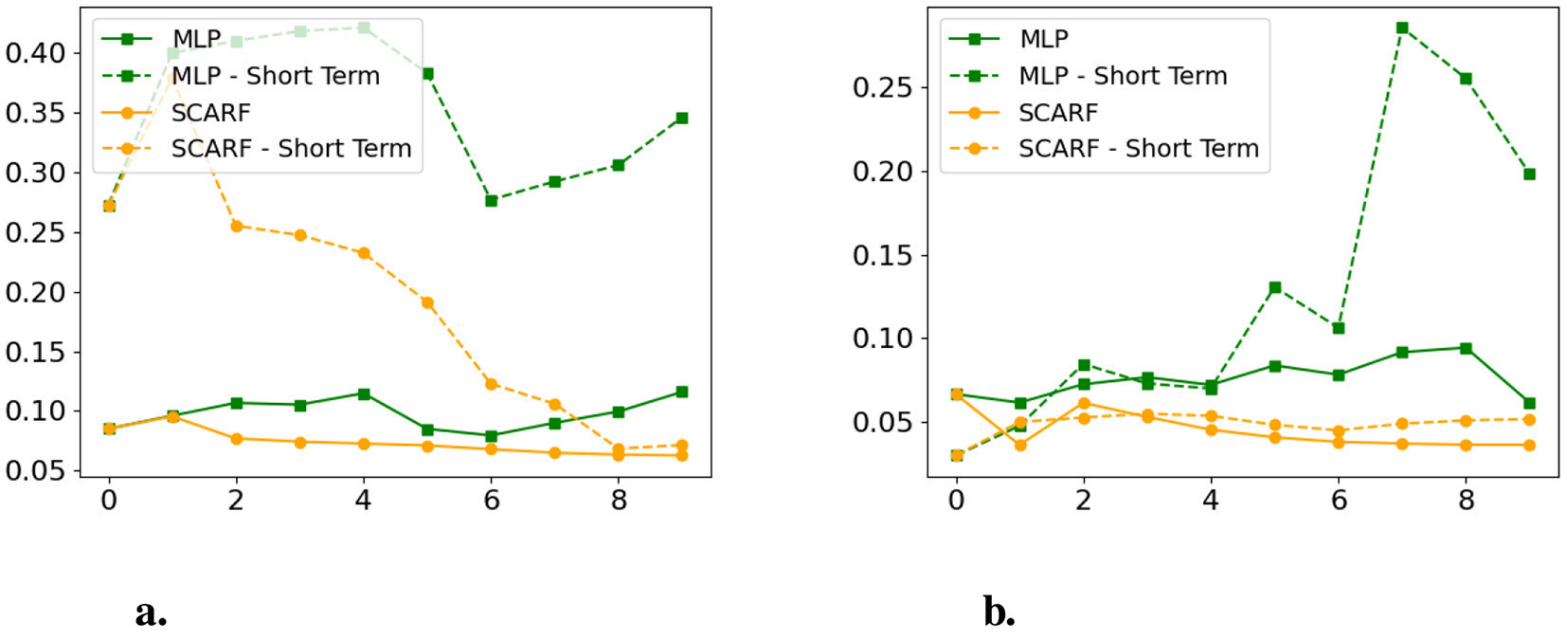
Ablation study on the LSTM intervention generator. X-axis: time step; Y-axis: fairness discrepancy (lower is better). **(a)** Synthetic dataset. **(b)** Taiwan dataset.

Lastly, we perform an ablation study to understand the role of the LSTM-based sequential intervention module within SCARF. Specifically, we compare the original SCARF framework (with LSTM) to a variant that uses a multilayer perceptron (MLP) to generate interventions, given identical initial conditions and constraints. Figure 9 shows that the LSTM-based variant exhibits lower fairness discrepancies over time in both short-term and long-term measures. This comparison highlights the importance of modeling temporal dependencies and incorporating information from past interventions when generating sequential recourse trajectories.

### 5.4 Discussion and limitations

The experimental results illustrate how temporally structured recourse can mitigate long-term unfairness relative to myopic or single-step alternatives. We emphasize that SCARF is not designed to characterize optimal or adaptive intervention schedules. Identifying such policies would require solving a constrained sequential decision-making problem under causal uncertainty, which is beyond the scope of this work. Instead, SCARF serves as a causally grounded reference framework that makes temporal feasibility and long-term fairness explicit and empirically analyzable.

## 6 Conclusions

In this paper, we studied the problem of sequential algorithmic recourse under long-term fairness considerations and introduced Sequential Causal Algorithmic Recourse for Fairness (SCARF) as a causally grounded framework for generating temporally coherent recourse trajectories. Unlike prior approaches that pursue long-term fairness by modifying decision models directly, SCARF operates at the level of individual interventions, producing actionable and personalized recommendations while preserving the underlying decision policy. By integrating structural causal modeling with sequential generative modeling, SCARF provides a concrete instantiation of how causal dependencies and temporal dynamics can be incorporated into recourse generation. Through experiments on synthetic and semi-synthetic datasets, we empirically examined how different recourse strategies influence the trade-offs between short-term and long-term fairness over time. Overall, this work highlights the importance of explicitly modeling temporal structure and causal constraints when studying algorithmic recourse in dynamic settings. We hope that SCARF serves as a useful reference framework for future research on sequential recourse, long-term fairness, and causal decision-making.

## Data Availability

The original contributions presented in the study are included in the article/supplementary material, further inquiries can be directed to the corresponding author.
